# Parameter-Determined Effects: Advances in Transcranial Focused Ultrasound for Modulating Neural Excitation and Inhibition

**DOI:** 10.3390/bioengineering13010020

**Published:** 2025-12-25

**Authors:** Qin-Ling He, Yu Zhou, Yang Liu, Xiao-Qing Li, Shou-Kun Zhao, Qing Xie, Gang Feng, Ji-Xian Wang

**Affiliations:** 1 School of Gongli Hospital Medical Technology, University of Shanghai for Science and Technology, Shanghai 200093, China; asteriahql@163.com; 2 Department of Rehabilitation Medicine, Ruijin Hospital, School of Medicine, Shanghai Jiao Tong University, Shanghai 200025, China; zhouy4595@163.com (Y.Z.); ly12446@rjh.com.cn (Y.L.); 15253622398@163.com (X.-Q.L.); zsk01n10@rjh.com.cn (S.-K.Z.); ruijin_xq@163.com (Q.X.); 3 Department of Rehabilitation Medicine, Shanghai Ruijin Rehabilitation Hospital, Shanghai 200023, China; 4 Department of Critical Care Medicine, Shanghai Pudong New Area Gongli Hospital, No. 219, Miaopu Road, Pudong New Area, Shanghai 200135, China

**Keywords:** transcranial focused ultrasound stimulation, neuromodulation, ultrasound parameters, excitability, inhibition, safety

## Abstract

Transcranial focused ultrasound stimulation (tFUS), an emerging non-invasive neuromodulation technique, has garnered growing attention owing to its high spatial resolution and precise targeting capability for deep brain structures. A body of evidence demonstrates that tFUS can effectively modulate neural activity in specific brain regions, inducing excitatory or inhibitory effects, and it is an important means to reshape neural functions. Ultrasound parameters are crucial in determining the transcranial ultrasound modulation effects. However, there is still controversy over which parameters can regulate neural excitability or inhibition, and there are significant differences in the parameters used in previous studies, which have limited the clinical application of transcranial ultrasound to some extent. Therefore, a systematic clarification of parameter–effect relationships is urgently needed to enable qualitative and quantitative understanding of ultrasound-induced neuromodulation, which is essential for achieving reliable and reproducible outcomes. This paper intends to review the effects of different tFUS parameters and their combinations on the excitability and inhibition of brain neural activities as well as the possible mechanisms. By integrating recent findings from both animal models and human clinical studies, we also discuss critical safety issues related to tFUS, aiming to provide a theoretical basis for future transcranial focused ultrasound modulation treatments for various neurological diseases such as stroke, Parkinson’s disease, dementia, epilepsy, pain disorders, and disorders of consciousness while providing reference value for selecting tFUS treatment regimens.

## 1. Introduction

Transcranial focused ultrasound (tFUS) has emerged as a cutting-edge and rapidly evolving technology in neuroscience and rehabilitation medicine. Compared to conventional non-invasive neuromodulation techniques such as transcranial magnetic stimulation and transcranial electrical stimulation, tFUS offers superior spatial resolution and the unique capability to precisely target deep brain structures [1,2]. Accumulating evidence indicates that tFUS not only modulates local neuronal electrical activity but also exerts therapeutic effects by regulating neural networks, highlighting its promising potential for treating a wide range of neuropsychiatric disorders [3,4].

The development of tFUS aims to overcome the inherent limitations of current mainstream neural modulation techniques. Traditional non-invasive approaches such as TMS and tDCS are constrained by their physical principles, leading to a fundamental “depth-focality trade-off”: TMS predominantly influences superficial cortical layers and struggles to effectively engage deep brain structures [5], whereas tDCS produces broad and diffuse electric field distributions, resulting in limited spatial resolution [6]. Consequently, both modalities lack the precision required to selectively modulate deep neural circuits—such as those within the limbic system and basal ganglia—that are critically implicated in disorders like depression and Parkinson’s disease. Although deep brain stimulation (DBS) can effectively target these subcortical regions, its invasive nature entails significant surgical risks and high treatment costs, which restrict its broad clinical adoption [7]. In this context, tFUS emerges as a promising alternative, combining non-invasiveness with high spatial resolution and robust depth penetration [2]. The acoustic energy delivered by tFUS can traverse the skull and generate focal points on the order of millimeters within deep brain areas, enabling precise neuromodulation of subcortical targets [2]. Accumulating evidence indicates that tFUS can safely and reversibly modulate neuronal excitability in target-ed areas, achieving spatial accuracy markedly superior to conventional non-invasive methods [8]. As such, tFUS is increasingly recognized as a transformative technology capable of overcoming the dual challenges of targeting depth and spatial specificity, thereby offering novel avenues for the precise intervention of treatment-resistant neurological and psychiatric conditions [7,9].

In recent years, tFUS has shown promising potential in exploratory studies targeting a range of neurological disorders. In psychiatric conditions, tFUS can alleviate depressive and anxiety symptoms through modulation of deep limbic nuclei, including the amygdala [10]. For Parkinson’s disease-related motor impairments, it enables non-invasive and spatially accurate regulation of motor-associated brain regions such as the subthalamic nucleus [9]. In chronic pain management, tFUS modulates pain perception by targeting critical cortical areas, including the primary somatosensory cortex and insula [11]. In patients with disorders of consciousness, tFUS enhances thalamocortical functional connectivity, offering a novel therapeutic avenue for promoting arousal and recovery [12]. Furthermore, in stroke rehabilitation, neuromodulation of the sensorimotor cortex via tFUS is increasingly recognized as a mechanism that supports neural plasticity and functional re-organization [13].

Neural excitability constitutes a fundamental basis for brain function regulation and neural plasticity. tFUS can significantly modulate neural plasticity by regulating the balance between ex-citation and inhibition in targeted brain regions, positioning it as a promising tool for neuromodulation and neurorehabilitation [10]. Nevertheless, tFUS remains in the early stages of clinical translation, with its optimal stimulation parameters, long-term therapeutic effects, and underlying neurobiological mechanisms yet to be fully elucidated—requiring rigorous validation through large-scale, well-controlled clinical studies [10,12]. A major challenge currently limiting its application lies in the complexity of core parameter selection—including ultrasound frequency, duty cycle, pulse repetition frequency, sonication duration, and intensity—where diverse combinations may elicit distinct neural responses, and no standardized protocols have been established [3,7]. Moreover, even when identical stimulation parameters are applied, discrepancies in outcomes related to neural excitability and inhibitory modulation have been reported across studies [14]. This variability in parameter implementation not only impedes a systematic under-standing of tFUS mechanisms but also hampers its reproducibility and broad adoption in both research and clinical settings.

**Figure 1:**
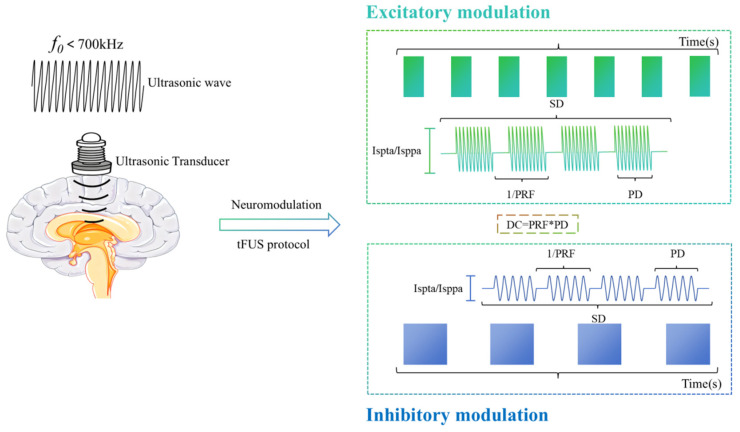
Core parameters of transcranial focused ultrasound and its neuromodulation effects. The *f*_0_ < 700 kHz helps reduce attenuation during its propagation in the cranium, thereby penetrating the skull more effectively and acting on the brain target area. The height of the line segment represents Ispta/Isppa, the width of the square reflects SD, the density of the ultrasound waveform corresponds to the PRF, and DC = PRF × PD. Among them, excitatory modulation uses the parameter combination of “high intensity, high PRF, high DC, and short SD”; inhibitory modulation, on the other hand, adopts the parameter combination of “low intensity, low PRF, low DC, and long SD”.

Accordingly, this review aims to comprehensively summarize the influence of various tFUS parameters on the modulation of neural excitability and inhibition (Figure 1). The methodological details of the literature search are provided in Appendix A. By synthesizing recent findings from animal models and human studies, we examine the roles and potential mechanisms through which different parameter configurations affect neural activity and plasticity. The goal is to provide a theoretical basis and parameter references for optimizing tFUS treatment protocols and ensuring safety in clinical applications; thereby promoting standardized application of this technology in the neuromodulation field.

## 2. Core Parameters of tFUS and Their Effects on Neuromodulatory Effects

The implementation of tFUS stimulation depends on the precise design of ultrasound transducers, with their focusing capability, penetration depth, and safety governed by key transducer parameters and underlying core technologies (see Appendix B). The neuromodulatory effects of tFUS are mediated by a set of interdependent physical parameters that collectively determine the efficiency of cranial transmission, spatial targeting accuracy, and the resulting neural excitation or inhibition. Among these, ultrasound frequency plays a pivotal role in balancing tissue penetration depth with spatial resolution; frequencies ranging from 200 to 650 kHz are typically employed to achieve effective transcranial delivery. Additional critical parameters—including Duty Cycle, Pulse Repetition Frequency, Sonication Duration, and intensity [15], etc.—determine the physiological effects of ultrasound on neurons after it enters the brain. Notably, variations in individual parameters or their combinations can lead to distinct neuromodulatory outcomes [16,17].

### 2.1. Ultrasonic Frequency (Frequency, f_0_)

The ultrasonic frequency (*f*_0_) refers to the number of complete periodic oscillations per unit time and is inversely proportional to the wavelength. In transcranial ultrasound therapy, *f*_0_ critically influences both the depth and spatial precision of neuromodulatory effects. Lower frequencies correspond to longer wavelengths, enabling deeper penetration but yielding lower spatial resolution. Conversely, higher frequencies produce shorter wavelengths and smaller focal volumes, allowing for finer spatial targeting. When *f*_0_ exceeds 1 MHz, the focal spot of ultrasonic stimulation can be confined to a few millimeters [18,19]. However, with increasing frequency, ultrasound energy attenuates more rapidly as it traverses heterogeneous media such as the skull, thereby limiting penetration depth [19].

For effective neural modulation using tFUS, sufficient acoustic energy must reach the target brain parenchyma, necessitating careful selection of *f*_0_ [20]. In vitro studies on human skulls have demonstrated that frequencies below 700 kHz are optimal for transcranial transmission efficiency [18,21,22,23]. Consequently, most tFUS studies in non-human primates and humans employ transducer frequencies within the 200–650 kHz range. At these frequencies, spatial resolution permits targeting of brain regions measuring 2–4 mm in diameter [22,24,25]. Thus, the role of *f*_0_ in tFUS-mediated neuromodulation primarily involves optimizing the trade-off between cranial penetration and spatial localization, rather than directly determining whether neural activity is excited or inhibited.

### 2.2. Ultrasonic Duty Cycle (DC)

The duty cycle (DC) of ultrasonic waves refers to the proportion of effective stimulation time within a single acoustic cycle and directly determines the average acoustic power and thermal effects. Higher DC values increase the likelihood of tissue heating, potentially leading to localized damage; therefore, careful selection of DC is essential in transcranial ultrasound therapy. Although continuous ultrasound (DC = 100%) has been shown to induce neuronal electrical activity, pulsed ultrasound—characterized by non-continuous, intermittent delivery—is typically preferred due to the low activation threshold of neurons [26,27] and the need to minimize thermal accumulation in brain tissue [28,29].

DC is a critical parameter influencing whether neuromodulatory outcomes are excitatory or inhibitory. According to the Neuronal Intracellular Cavitation Excitation (NICE) model, Plaksin et al. demonstrated that low DC (<5%) preferentially activates thalamic reticular neurons (TRN), thalamocortical neurons (TCN), and low-threshold spiking interneurons (LTS) via T-type voltage-gated calcium channels, resulting in net inhibitory effects. In contrast, high DC (>20%) predominantly elicits excitatory responses by activating regular spiking pyramidal cells (RS) and fast spiking interneurons (FS), while simultaneously suppressing LTS interneuron activity [30]. Yoon et al. applied tFUS to the sheep brain and observed that stimulation of the primary motor cortex (M1) and thalamus at high DC levels (30%, 50%, 70%, 100%) induced excitatory effects, with maximal efficacy achieved at 70% DC under otherwise identical parameters. Conversely, low DC (3–5%) stimulation of the primary somatosensory cortex (S1) and thalamus produced inhibitory responses [31]. Similarly, Kim et al. reported in rats that 50% DC applied to M1 yielded stronger neural excitation compared to 30% and 70% DC [29]. Collectively, these findings indicate that DC not only governs safety-related thermal considerations but also plays a pivotal role in determining the direction—excitation or inhibition—of neuromodulatory effects in tFUS applications.

### 2.3. Pulse Repetition Frequency (PRF)

Pulse Repetition Frequency (PRF) refers to the number of ultrasonic pulse trains delivered per second. To achieve effective neuromodulation, a higher PRF is typically employed to increase the average acoustic energy delivered to neural tissue. PRF is also a key parameter in determining whether transcranial ultrasound induces excitatory or inhibitory effects. Yu et al. applied tFUS to S1 of anesthetized mice and demonstrated intrinsic differences in how excitatory and inhibitory neurons respond to PRF. High-frequency PRF tFUS robustly activates excitatory neurons, with neuronal excitability increasing in a dose-dependent manner—even when the DC is held constant. In contrast, inhibitory neurons are insensitive to PRF, and low-frequency PRF has no significant effect [32].

Further evidence supports the frequency-dependent neuromodulatory role of PRF. In studies using different PRF values (30 Hz, 140 Hz, 500 Hz) to stimulate the mouse M1, 500 Hz stimulation significantly enhanced the amplitude of motor evoked potentials (MEPs) [33]. Similarly, high-frequency PRF (2000 Hz) tFUS applied to rat M1 resulted in a marked increase in MEP amplitude [34]. In humans, Legon et al. reported that tFUS stimulation of S1 at 500 Hz significantly amplified somatosensory evoked potential (SEP) amplitudes [8]. Yu et al. further showed that when applied to the lower limb motor area, high PRF (3000 Hz) more effectively enhanced motor-related cortical potentials (MRCPs) compared to low PRF (300 Hz) [35]. Conversely, Zadeh et al. found that low PRF stimulation of human M1 produced inhibitory effects: a 10 Hz PRF significantly reduced MEP amplitude, with suppression lasting at least 30 min, while 100 Hz induced inhibition persisting up to 60 min [36]. Additionally, Badran et al. observed that two 10 min sessions of anterior thalamic tFUS (PRF = 10 Hz; DC = 5%) significantly reduced thermal pain sensitivity in healthy individuals [37].

Collectively, these findings indicate that high-frequency PRF (>100 Hz) tends to elicit excitatory neuromodulatory effects, whereas low-frequency PRF (5–100 Hz) is more likely to induce sustained inhibitory responses.

### 2.4. Sonication Duration (SD)

SD refers to the total duration from the onset of the first pulse to the termination of the last pulse, and it directly determines the total amount of ultrasonic energy delivered to the target tissue. Accumulating evidence indicates that SD plays a critical role in modulating neural activity, with distinct effects on neuronal excitation or inhibition depending on its duration. Tyler et al. demonstrated in both in vitro (hippocampal slices) and in vivo (anesthetized rats) experiments that short-duration pulses (SD < 100 ms) in transcranial focused ultrasound stimulation (tFUS) can effectively elicit neuronal action potentials [38]. Similarly, King et al. reported that brief SD pulses (~300 ms) rapidly activated neurons in the rat motor cortex, leading to observable limb movements [39]. Tufail et al. further confirmed in mice that short SD stimuli (<500 ms) reliably induced excitatory responses in both the motor cortex and hippocampus [26]. In contrast, Yoo et al. applied tFUS with a prolonged SD (9 s) to the primary visual cortex (V1) of rabbits and observed an 80% reduction in the amplitude of the P30 component of the visual evoked potential (VEP), along with a 30% decrease in the peak blood oxygen level-dependent (BOLD) signal [40]. Additionally, Yoon et al. found that extended SDs (~1 min) at low duty cycles (≤10%) predominantly elicited inhibitory neural effects [31]. Collectively, these findings suggest that millisecond-scale SDs are generally associated with neuronal excitation, whereas longer SDs tend to induce inhibitory outcomes.

### 2.5. Ultrasound Intensity (I)

Ultrasound intensity is commonly characterized by two key parameters: space-peak-time-average intensity (Ispta) and space-peak-pulse-average intensity (Isppa). Ispta measures the average intensity throughout the entire ultrasound treatment period, reflecting the risk of thermal effects under long-term exposure and being directly proportional to DC. Isppa measures the average intensity of a single pulse, reflecting the peak sound intensity during a single pulse or short-term exposure. According to the NICE model [30], under low DC conditions, low Isppa values (0.1–1 W/cm^2^) preferentially activate highly sensitive neurons such as LTS cells and TRN/TCN populations. Activation of LTS neurons enhances inhibitory synaptic transmission, thereby strongly suppressing the firing of excitatory cortical neurons, including RS types. Conversely, at high DC, elevated Isppa (>1 W/cm^2^) activates less sensitive neuronal populations, such as RS and FS neurons. Under these conditions, LTS neurons are suppressed due to sustained hyperpolarization, which shifts network dynamics toward increased cortical excitation.

Ultrasound can modulate cellular activity by activating mechanosensitive ion channels. In vitro studies have demonstrated that ultrasound stimulation opens the K^+^-selective mechanosensitive channel TRAAK, with the magnitude of induced current increasing as Isppa rises [41]. However, this relationship is nonlinear; beyond a certain threshold, the effect plateaus or may even reverse. For instance, thalamic calcium responses saturate when Ispta exceeds 5.4 W/cm^2^ [42], and intensities above 3.7 W/cm^2^ can elicit inhibitory neural responses evidenced by reduced SEP amplitudes [43]. These inhibitory effects may arise from thermal mechanisms or ultrasound-induced neuronal hyperpolarization. Kim et al. investigated the effects of 8 MHz transcranial focused ultrasound on the mouse somatosensory cortex using two distinct Ispta levels (468 mW/cm^2^ and 1077 mW/cm^2^), observing that hemodynamic response amplitudes increased with higher acoustic intensity, indicating a positive relationship between Ispta and neurovascular coupling [44]. Therefore, careful selection of ultrasound intensity is critical in neuromodulation: high Isppa must be tightly controlled to avoid transient pressure-induced neuronal damage while excessive Ispta can lead to localized tissue heating [45]. As such, ultrasound intensity is also a pivotal parameter in determining whether transcranial ultrasound induces excitatory or inhibitory neural outcomes.

## 3. Parameter Combination Schemes of tFUS and Neuromodulatory Effects

The neuroregulatory effects of tFUS, including neuronal excitation or inhibition, are not governed by any single parameter but result from the synergistic interaction of key acoustic parameters such as *f*_0_, DC, PRF, SD, and intensity. A synthesis of evidence from multiple animal and human studies reveals that excitatory neuromodulation is typically associated with parameter combinations involving high intensity, high DC, high PRF, and short SD (Table 1), whereas inhibitory effects are predominantly elicited by low intensity, low DC, low PRF, and long SD (Table 2). Multiple studies have optimized tFUS parameter combinations to achieve excitatory modulation of brain regions in the central nervous system; neural activation or functional enhancement effects have been observed in various experimental models. tFUS can not only modulate cortical regions but also effectively modulate deep brain tissues. This modulatory effect can simultaneously affect the target area and tissues outside the target area while showing potential in modulating motor function, higher cognitive function, pain perception, and abnormal neuronal discharges.

### 3.1. Excitatory Modulation Protocol of tFUS

In preclinical animal studies, Fisher and Gumenchuk et al. applied tFUS to the S1 of healthy mice, utilizing voltage-sensitive dyes (VSDs) to measure cortical electrical activity evoked by somatosensory stimuli and monitoring calcium dynamics in GCaMP6f-expressing transgenic mice. Their findings demonstrated that tFUS advanced the onset latency of sensory-evoked cortical responses by 3.0 ± 0.7 milliseconds and enhanced spatial stability of Ca^2+^ signaling in the forelimb region of S1 [46]. Baek et al. reported that tFUS stimulation of the lateral cerebellar nucleus in stroke-model mice elicited MEPs in the forelimbs [47]. Manuel et al. applied tFUS to brain slices containing the motor cortex from GCaMP6s transgenic mice and observed a significant increase in neuronal calcium signal responsiveness [48]. Li et al. demonstrated that tFUS targeting the hippocampus in Alzheimer’s disease mice improved impaired neurovascular coupling and normalized neural oscillatory patterns [49]. Yu et al. found that tFUS stimulation of the prefrontal cortex in normal rats induced focal activation at the target site, with subsequent spatiotemporal propagation of activation to adjacent cortical regions [50]. Hsieh et al. showed that intermittent tFUS applied to M1 in healthy rats not only augmented MEP amplitude but also elevated expression levels of c-Fos, a marker of neuronal activation [51]. Yang et al. further demonstrated that tFUS delivered to the somatosensory cortex of non-human primates activated both targeted and functionally connected non-targeted neuronal populations [52].

In human studies, tFUS has also demonstrated significant potential for neural modulation, as evidenced by an increasing number of investigations involving healthy adult participants. Lee et al. demonstrated that tFUS stimulation of the S1 cortex can elicit finger-specific tactile percepts and evoke SEP-like electrophysiological responses [53,54]. Mueller et al. reported that tFUS modulates phase dynamics within beta-band oscillations in the S1 region [55]. Both Lee et al. and Nandi et al. confirmed that tFUS stimulation of the V1 area could induce phosphenes or enhance VEPs [56,57]. Ai et al. applied tFUS to stimulate the M1 area and found a significant activation of the BOLD signal in the thumb—representing area of M1, while there were no obvious changes in other adjacent brain regions [58]. Zhang et al. found that applying excitatory tFUS to stimulate the motor cortex significantly increased the MEP amplitude, reduced short-interval intracortical inhibition (SICI) and long-interval intracortical inhibition (LICI), and significantly decreased the GABA concentration and increased the glutamate/glutamine (Glx) concentration [59]. Butler et al. applied tFUS to stimulate the human middle temporal complex (hMT+, also known as V5 area) and found that it improved the accuracy in the visual motion detection task and reduced the reaction time. Additionally, tFUS also caused changes in event-related potentials (ERPs), and the amplitude of these changes was correlated with the improvement in accuracy during the visual motion detection task [60]. Fine et al. used tFUS to activate the right inferior frontal gyrus (rIFG) (related to inhibitory control and behavioral flexibility) and found that it could improve the inhibitory effect by influencing the speed of the stopping process, and observed a significant shortening of the onset latency of N200/P300 [61]. Kim et al. applied tFUS to target S1 and its thalamic projection, the ventral posterolateral nucleus (VPL), successfully inducing electrical activity. They also found a significant enhancement of the functional connectivity between S1 and other cortices, as well as the extensive cortical and subcortical network connections of the VPL [62]. Excitingly, Monti et al. applied tFUS to stimulate the thalamus of a patient with disorders of consciousness, and three days later, successfully helped the patient emerge from the minimally conscious state [63].

Collectively, evidence across species—from rodents to non-human primates—and conditions—including healthy and disease model animals as well as healthy humans and clinical patients—supports the excitatory neuromodulatory effects of tFUS. These findings suggest that tFUS may serve as a promising intervention for enhancing sensorimotor function and cognitive processing, facilitating recovery from disorders of consciousness.

### 3.2. Inhibitory Modulation Protocol of tFUS

In the inhibitory research of tFUS, most studies have focused on humans, and there are relatively few animal experiments. Nevertheless, preclinical evidence supports its neuromodulatory potential. Yang et al. demonstrated that tFUS stimulation of the hippocampal CA3 region in a mouse model of temporal lobe epilepsy significantly prolonged seizure latency and reduced seizure duration [64]. Similarly, Min et al. reported that tFUS targeting the thalamus in rat models of epilepsy markedly suppressed epileptiform EEG bursts and attenuated behavioral seizure activity [65].

In healthy human participants, multiple studies have consistently documented inhibitory neurophysiological responses following tFUS application. Zadeh et al. and Fomenko et al. independently showed that low PRF tFUS applied to M1 significantly suppressed MEPs and enhanced SICI [36,66]. Zhang et al. further confirmed these inhibitory effects by reporting reduced MEP amplitudes, prolonged SICI and LICI, and decreased levels of Glx—a key excitatory neurometabolite—following inhibitory tFUS over M1 [59]. Additionally, Ziebell et al. found that tFUS stimulation of the right prefrontal cortex (PFC) attenuated mid-frontal theta oscillations, suggesting modulation of cognitive control networks [67].

Notably, tFUS has also demonstrated inhibitory effects when targeting deep brain structures. Badran et al. applied tFUS to the right anterior thalamus and observed a significant increase in thermal pain threshold, indicating a measurable analgesic effect [37]. Cain et al. applied tFUS to stimulate the left globus pallidus (GP) and found a significant decrease in BOLD signals in the local target area of GP and the distal large-scale cortical network, and a general decrease in the relative cerebral blood perfusion throughout the brain was observed [68].Clinically, Lee et al. applied tFUS to the seizure onset zone (SOZ) in patients with drug-resistant epilepsy (DRE), successfully modulating the stereoelectroencephalographic (SEEG) power spectrum and reducing seizure frequency in several individuals—providing preliminary yet compelling evidence for the therapeutic potential of tFUS in neurological disorders [69].

In summary, tFUS can achieve inhibitory effects on different brain regions and has shown certain potential in the treatment of movement disorders, pain, and epilepsy, providing new insights for the treatment of these diseases.

### 3.3. Parameter Strategies and the Mechanistic Transition Underlying tFUS-Induced Offline Effects

The neuromodulatory effects of tFUS extend beyond the immediate “online effects” observed during stimulation. Of greater translational significance are the “offline effects,” which persist from tens of minutes to hours after stimulation ceases. This capacity marks tFUS’s pivotal transition from a transient neuromodulatory tool to an intervention capable of inducing sustained neural plasticity.

Online effects primarily stem from the direct, reversible mechanical perturbation of neuronal membrane potentials by ultrasound [70]. In contrast, offline effects represent a distinct paradigm, mediated by the activation of endogenous neural plasticity pathways [71,72]. As synthesized from Table 1 and Table 2, the induction of offline effects—whether excitatory or inhibitory—relies on parameter strategies specifically designed to systematically “drive plasticity,” involving a mechanistic transition from rapid mechanotransduction to slower molecular cascades.

#### 3.3.1. From Transient Modulation to Sustained Remodeling: Parameter Strategies for Offline Effects

While the basic parameter trends (e.g., high PRF/DC for excitation) align with online effects, studies successfully eliciting sustained functional changes adopt more refined and systematic parameter designs. For excitatory offline remodeling, a “high-frequency rhythmic encapsulation” strategy is common. For instance, in stroke models, short stimulation with high PRF (1000 Hz) and moderate DC (50%) promotes long-term sensorimotor recovery [47]. In the human motor cortex, 2000 Hz PRF with 40% DC enhances MEP amplitude for a prolonged period [59]. The most prominent protocol is theta-burst ultrasound stimulation, which delivers pulses at a 5 Hz theta rhythm. Given the well-established role of theta oscillations in learning and plasticity, this protocol reliably induces LTP-like plasticity in the human M1, enhancing corticospinal excitability for over 30 min [73,74]. Recent studies have extended these findings by demonstrating that its effects are mediated by NMDA receptor-dependent mechanisms and mechanosensitive ion channels in the human motor cortex [75], are associated with GABAergic modulation and prolonged functional connectivity alterations in deep cortical networks [76], and can induce Akt-dependent metaplasticity that persistently modifies hippocampal circuit function [77].

For inhibitory offline remodeling, a “low-frequency sustained accumulation” strategy is often employed. Zadeh et al. used 250 kHz, 10/100 Hz PRF, 10% DC, and a 120 s duration to induce MEP suppression lasting 30–60 min in healthy volunteers [36]. Badran et al. applied 650 kHz, 10 Hz PRF, 5% DC, and 30 s stimulation to the anterior thalamus, yielding a persistent analgesic effect [37]. In epilepsy models, low PRF (100–500 Hz), low DC (5%), and longer durations effectively suppress seizures [64,65].

These findings indicate that successful offline remodeling requires not only sufficient stimulus energy but, crucially, a temporal structure that resonates with the brain’s intrinsic plasticity mechanisms, efficiently triggering lasting changes from synapses to circuits.

#### 3.3.2. Mechanistic Hub: From Mechanical Gating to Molecular Plasticity

The temporal divergence between online and offline effects originates from fundamentally different mechanisms. Online effects are driven by the rapid ultrasound activation of mechanosensitive ion channels (e.g., Piezo1, TRP channels), causing transient Na^+^/Ca^2+^ influx and membrane potential shifts [78]. These effects cease promptly with stimulation. Offline remodeling entails a delayed, multi-stage molecular cascade:

① Calcium Signaling: Initial Ca^2+^ influx acts as a core second messenger, activating kinases like CaMKII and PKA [26].

② Synaptic Plasticity Pathways: These kinases phosphorylate and modulate AMPAR and NMDAR, laying the molecular ground-work for LTP or LTD [8].

③ Gene Expression: Sustained or patterned stimulation activates transcription factors (e.g., CREB), upregulating plasticity-related genes (e.g., BDNF, c-Fos) that support synaptic growth and stability [47,51].

④ Glial Modulation: Ultrasound-sensitive astrocytes release gliotransmitters (e.g., ATP, D-serine), which can modulate synaptic strength and plasticity over extended periods [79,80].

Thus, effective offline remodeling strategies are those whose temporal parameters optimally convert exogenous mechanical energy into enduring endogenous molecular signals for sustained circuit-level changes.

In summary, the programmability of tFUS parameters enables not only immediate control of neural excitability but also, through deliberate parameter strategies, facilitates a critical transition from transient modulation to sustained, plasticity-mediated functional remodeling.

## 4. Safety of tFUS

The selection of ultrasound parameters is a core factor determining the safety of tFUS. Different combinations of ultrasound parameters can elicit distinct neurophysiological responses. Therefore, the appropriate selection of these parameters is a prerequisite for ensuring safety. Among these parameters, the Mechanical Index (MI) serves as a key metric for evaluating the risk of potentially harmful biomechanical effects—such as inertial cavitation—in biological tissues [81], with its calculation being directly dependent on acoustic pressure. Acoustic pressure, measured in megapascals (MPa), represents a core physical parameter that characterizes the mechanical force exerted by ultrasound waves and can directly influence neuronal activity. However, no consistent correlation has been established between specific levels of acoustic pressure and neuroregulatory outcomes (i.e., inhibitory versus excitatory effects). Evidence indicates that identical acoustic pressures (e.g., 0.13 MPa) may induce excitatory responses in certain neurons while producing negligible effects in others within the same brain region [82]. Similarly, under comparable pathological conditions, different pressure levels (e.g., 0.35 MPa and 0.13 MPa) have both been associated with inhibitory outcomes [83]. These findings underscore the highly context-dependent nature of acoustic pressure’s biological effects, which are modulated by factors such as neuronal subtype, local neural circuitry, and interactions with other ultrasound parameters. Consequently, careful control of acoustic pressure and its derived MI value is essential to mitigate the risk of mechanical tissue damage.

A recent clinical trial investigating tFUS for treatment-resistant substance use disorders reported an adverse event involving brain injury, prompting significant attention and scholarly debate [84]. In-depth review revealed that the acoustic parameters employed—specifically, an estimated intracranial peak negative pressure of 2.1 to 3.5 MPa, corresponding to an MI of approximately 3.4 to 4.0—substantially exceeded established safety thresholds for low-intensity neuromodulation, potentially placing the intervention within the medium- to high-intensity range. More critically, the study lacked full disclosure of key safety-related parameters, including detailed spatial mapping of the intracranial acoustic field and assessments of thermal accumulation, thereby impeding independent validation and replication efforts [85]. This incident underscores the dual importance of accurately controlling and comprehensively reporting actual intracranial acoustic exposure levels—including acoustic pressure, intensity, and MI—as these practices are not only central to ensuring patient safety but also foundational to the reproducibility and scientific credibility of research findings. In subsequent open communications, multiple experts clarified that this case does not invalidate the overall safety profile of low-intensity tFUS when conducted within recognized safety limits (e.g., MI < 1.9). They further emphasized that adherence to such thresholds, coupled with improved transparency in parameter reporting, is vital for distinguishing safe neuromodulatory applications from potentially hazardous interventions and for fostering consensus within the scientific community [86].This controversial case and the ensuing discourse collectively highlight the necessity of establishing clear, widely accepted safety boundaries for acoustic exposure and rigorously enforcing them in clinical and research settings to ensure the responsible advancement of low-intensity neural modulation technologies.

In addition to mechanical effects, thermal effects represent another critical aspect of safety assessment in transcranial ultra-sound applications. Given that both mechanical and thermal mechanisms have the potential to induce brain tissue damage, the U.S. Food and Drug Administration (FDA) guidelines for cranial ultrasound recommend limiting ultrasound exposure parameters to the following thresholds to ensure safety: Ispta < 94 mW/cm^2^, Isppa < 190 W/cm^2^, and MI < 1.9. These limits are established to minimize the risks of adverse bioeffects, including inertial cavitation and excessive thermal accumulation [87]. Additionally, to promote consistency and reproducibility across studies, the International Transcranial Ultrasound Stimulation Safety and Standards Consortium (ITRUSST) has developed standardized reporting guidelines for tFUS experiments. These guidelines emphasize comprehensive documentation of key components, including the ultrasound driving system, detailed acoustic parameters, pulse sequence design, and estimated intracranial exposure levels—enabling rigorous cross-study comparisons and robust safety evaluations [88,89]. From a technical standpoint, low-frequency ultrasound (*f*_0_ = 200–650 kHz) is commonly employed to enhance skull transmission efficiency while minimizing energy absorption and associated heat generation in overlying tissues [18,22,24,25]. Furthermore, the use of pulsed ultrasound modes significantly reduces the risk of thermal accumulation compared to continuous-wave delivery [29]. Similarly, shorter SD and lower PRF further contribute to thermal safety by limiting total energy deposition thereby mitigating potential thermal injury to neural tissue [90]. In practice, Isppa is typically maintained within the range of 0.5–31 W/cm^2^; numerous preclinical and human studies have reported no observed tissue damage when operating within these parameters [91,92].

A substantial body of evidence from both animal and clinical studies has confirmed that tFUS demonstrates a favorable safety record when following the above parameter guidelines. Histological analyses of animal experiments have shown that under effective stimulation parameters, no significant neuronal inflammation, apoptosis, or structural damage was detected [90,93]. Notably, a comparative study applying both intermittent and continuous tFUS to M1 in rats found no evidence of tissue injury or elevated inflammatory biomarkers in either mode [44]. Importantly, even with prolonged intervention—such as repeated tFUS administration over two months in spontaneously hypertensive rats—no histopathological brain damage was detected while sustained blood pressure regulation was achieved, underscoring the long-term safety and therapeutic potential of this modality [94].

In clinical studies, the safety of tFUS has been robustly validated across diverse protocols, ranging from single short-duration sessions (several minutes) to multiple longer-duration applications (e.g., up to 30 min per session). A comprehensive systematic review encompassing 677 participants—including both healthy volunteers and patient populations—reported an extremely low incidence of adverse events (approximately 3.4%), all of which were mild and transient. Common symptoms included headache, localized skin warming at the stimulation site, fatigue, or mild mood fluctuations, with complete resolution occurring shortly after stimulation ceased [95]. Similarly, a retrospective analysis of 120 individuals accepted for tFUS revealed no serious adverse events; any minor side effects—such as transient attentional lapses or muscle twitching—were fully reversible [96]. Critically, even in studies employing extended single-session durations of up to 30 min, no significant increase in adverse reactions was observed; no delayed or persistent neurological sequelae were reported during follow-up assessments [95,97]. These findings are further supported by thermal monitoring data at the skin-transducer interface which indicate that temperature elevation remains well within safe limits—typically less than 1.5 °C—under standard stimulation protocols [92].

In conclusion, current evidence strongly supports tFUS as a safe and reliable non-invasive neuromodulation modality when implemented within a scientifically optimized and standardized parameter framework. Adverse reactions are infrequent, mild, and self-limiting, with no credible reports of permanent tissue injury or enduring neurological deficits to date. Recent controversial cases serve as cautionary counterexamples: they demonstrate that exceeding established safety thresholds for low-intensity neurostimulation—such as MI values above 1.9—can markedly elevate risks. Thus, strict adherence to consensus-based safety limits and rigorous implementation of standardized parameter reporting are essential prerequisites for ensuring the continued safe translation of tFUS into clinical practice [85,86].

## 5. Conclusions

Transcranial focused ultrasound (tFUS), as a novel neuromodulation technology, is gradually revealing its unique potential in the domains of neuroscience and clinical medicine. Abundant clinical and animal experimental data suggest that within a reasonable parameter range, tFUS does not induce significant tissue damage or abnormal neurological function. By modulating key parameters including frequency, duty cycle, pulse repetition frequency, ultrasound duration, and acoustic intensity, tFUS can achieve bidirectional regulation of brain excitability and inhibition. This provides novel ideas and methods for the mechanism research and therapeutic interventions of various neuropsychiatric disorders. Existing research indicates that there exists a dynamic association between the selection of tFUS parameters and the neuromodulation effects. Specifically, the ultrasound frequency may influence the spatial selectivity of the target region. A high duty cycle and high pulse repetition frequency, combined with short-duration stimulation, tend to evoke excitatory neuromodulation. In contrast, low-intensity and low duty cycle stimulation over a long period may preferentially activate inhibitory neural circuits, thus achieving inhibitory regulation. Nevertheless, the above-mentioned effect patterns are not absolute, and the specific laws of action still require further systematic investigations for verification.

To objectively and quantitatively assess the neuromodulatory effects of tFUS, future research should incorporate multimodal neuroimaging and electrophysiological monitoring approaches. The shifts in cortical excitability and inhibitory states induced by tFUS are expected to manifest as changes in power and phase dynamics within specific EEG frequency bands—particularly alpha (α), beta (β), and gamma (γ) rhythms. These oscillatory activities are well-established neurophysiological markers of local neural network excitation-inhibition balance. For a comprehensive discussion on the physiological basis of each EEG rhythm, their association with neuronal excitation and inhibition, and their potential utility as biomarkers of tFUS-induced modulation, refer to Appendix C.

To further promote the development of tFUS into a safe, efficient, and reproducible neuromodulation approach, future endeavors should be dedicated to establishing a standardized parameter system, delving deeper into the exploration of its biological mechanisms, and validating its therapeutic efficacy through rigorous clinical trials. Eventually, the precise application of tFUS in clinical practice can be realized.

## Figures and Tables

**Table 1:** Excitatory modulation protocol of tFUS.

Study	Subjects	Target Regions	*f*_0_ (kHz)	PRF (Hz)	DC (%)	SD	Intensity (Measurement Conditions)	Acoustic Pressure (MPa)	Main Effects
Fisher et al. [46]	Normal mice (*n* = 17)	S1	510	1000	NR	1 s	Ispta = NR; Isppa = 0.69 W/cm^2^ (In degassed water)	0.17 MI: NR	Online: Shorten the latency of neural activity and influence the spatial pattern of calcium signals.
Baek et al. [47]	Stroke mice (*n* = 33)	LCN	350	1000	50	300 ms	Ispta = 2.54 W/cm^2^; Isppa = 1.25 W/cm^2^ (In degassed water)	NR MI: 0.54	Offline: Its intervention can effectively facilitate the long-term recovery of sensorimotor function following stroke and exert neuroprotective effects (e.g., alleviating cerebral edema).
Manuel et al. [48]	Brain sections of transgenic mice (*n* = 53)	M1	500	1500	60	0.4 ms	Ispta = NR; Isppa = NR (In degassed water)	0.1 MI: NR	Online: An increase in calcium signals was observed, with the response rate to 1500 Hz PRF pulsed ultrasound (29%) being significantly higher than those elicited by continuous wave (<15%) and 300 Hz PRF pulsed ultrasound (5%).
Li et al. [49]	AD mice (*n* = 24)	hippocampus	500	1000	30	50 ms	Ispta = NR; Isppa = NR (NR)	0.31 MI: NR	Offline: It enhanced the impaired cerebral blood oxygen metabolic response and improved neural oscillation patterns by suppressing theta waves and enhancing gamma wave activity.
Yu et al. [50]	Anesthetized rats (*n* = 3)	Prefrontal cortex	500	2000	NR	200 ms	Ispta = 0.1–0.6 mW/cm^2^; Isppa = 0.74–4.6 mW/cm^2^ (In degassed water)	0.0183–0.0459 MI: NR	Online: It induced time-locked EEG responses, and the response amplitudes were enhanced as the ultrasound intensity increased.
Hsieh et al. [51]	rat (*n* = 24)	M1	1000	NR	30	30 s	Ispta = 187.7 mW/cm^2^; Isppa = NR (In degassed water)	0.137 MI: NR	Offline: The amplitude of MEP increased, accompanied by upregulated c-Fos expression and downregulated GAD-65 expression.
Yang et al. [52]	Macaque (*n* = 2)	S1 (3a/3b)	250	2000	50	300 ms	Ispta = 20.9/94/446 mW/cm^2^; Isppa = 0.4/1.9/8.9 W/cm^2^ (Obtained through measurements in degassed water experiments with transcranial attenuation correction.)	0.925/0.425/0.2 MI: 0.68/1.0/1.46	Online: The BOLD signal in the S1 increased as stimulus intensity increased.
Lee et al. [53]	Healthy individuals (*n* = 18)	S1	250	500	50	300 ms	Ispta = 1.5 W/cm^2^; Isppa = 3 W/cm^2^ (Numerical simulations using Wave 3000 software)	NR MI: 0.62	Online: It elicits finger-specific tactile perception and responses analogous to somatosensory evoked potentials, which emerge and vanish in synchrony with the stimulus.
Lee et al. [54]	Healthy individuals (*n* = 10)	S1	210	500	50	500 ms	Ispta = 17.5 W/cm^2^ Isppa = 35 W/cm^2^ (In degassed water)	NR MI: NR	Online: Induce the tactile perception of the contralateral hand.
Mueller et al. [55]	Healthy individuals (*n* = 18)	S1	500	1000	36	0.5 s	Ispta = NR; Isppa = 23.87 W/cm^2^ (In degassed water)	0.8 MI: 1.13	Online: Modulate the phase dynamics of S1 neural oscillations within the Beta frequency band without changing the amplitude (power) of these oscillations.
Lee et al. [56]	Healthy individuals (*n* = 19)	V1	270	500	50	300 ms	Ispta = NR; Isppa = 0.7–6.6 W/cm^2^ (Numerical simulations based on individual CT and MRI data)	1.48 MI: 0.3–1.2	Online: Activate V1 and its associated neural network to elicit phosphenes. Specific evoked potentials emerge, namely N55 and P100.
Nandi et al. [57]	Healthy individuals (*n* = 14)	V1	270	250	50	300 ms	Ispta = NR; Isppa = 16 W/cm^2^ (In degassed water)	0.7 MI: NR	Online: Augmented the neural response (the N75 component of the VEP) elicited by external visual stimuli.
Ai et al. [58]	Healthy individuals (*n* = 15)	Thumb region, M1	500	1000	36	500 ms	Ispta = NR; Isppa = 16.95 W/cm^2^ (In degassed water)	NR MI: 0.97	Online: Augment the volume of BOLD activation during the finger-tapping task.
Zhang et al. [59]	Healthy individuals (*n* = 10)	M1	500	2000	40	500 ms	Ispta = 3.078–12.312 mW/cm^2^; Isppa = 0.6156–2.4624 W/cm^2^ (Measurements conducted in an ex vivo skull model)	NR MI: NR	Offline: The amplitude of MEP significantly increased; SICI and LICI significantly decreased; the proportion of Glx was upregulated.
Butler et al. [60]	Healthy individuals (*n* = 16)	hMT+, V5	500	1000	50	300 ms	Ispta = NR; Isppa = NR (Numerical simulations combining k-Wave software and human MRI data)	0.44 ± 0.12 MI: NR	Online: Enhance visual motion detection and modulate motion-induced ERP.
Fine et al. [61]	Healthy individuals (*n* = 63)	rIFG	500	1000	24	500 ms	Ispta = 5.38 W/cm^2^; Isppa = 22.43 W/cm^2^ (In degassed water)	0.82 MI: 1.15	Online: Enhance inhibitory performance and abbreviate the latency of P300.
Kim et al. [62]	Healthy individuals (*n* = 8)	S1; VPL	250	350/700/1400	70	200 ms	Ispta = NR; S1: Isppa = 4.1 ± 2.3 W/cm^2^ VPL: Isppa = 3.4 ± 1.1 W/cm^2^ (Measurements conducted in degassed water combined with numerical simulations based on individual CT data)	NR S1: MI = 0.7 ± 0.2 VPL: MI = 0.6 ± 0.1	Online: Evoke S1/thalamic potentials; Offline: Strengthen the functional connectivity of the sensorimotor region.
Monti et al. [63]	Individuals with disorders of consciousness (*n* = 1)	thalamus	650	100	NR	30 s	Ispta = 720 mW/cm^2^; Isppa = NR (NR)	NR MI: NR	Offline: After three days of treatment, the patient disengaged from the minimally conscious state.

**Table 2:** Inhibitory modulation protocol of tFUS.

Study	Subjects	Target Regions	*f*_0_ (kHz)	PRF (Hz)	DC (%)	SD	Intensity (Measurement Conditions)	Acoustic Pressure (MPa)	Main Effects
Yang et al. [64]	Epileptic mice (*n* = 5)	Hippocampus (CA3)	500	500	5	30 s	Ispta = 66.5 mW/cm^2^; Isppa = 1.75 W/cm^2^ (NR)	0.23 MI: 0.28	Online: Inhibit epileptic seizures.
Min et al. [65]	Epileptic rats (*n* = 27)	thalamus	690	100	5	NR	Ispta = 130 mW/cm^2^; Isppa = 2.6 W/cm^2^ (Obtained through measurements in degassed water experiments with transcranial attenuation correction)	0.27 MI: 0.33	Online: Inhibition of epileptic electrical activity and behavioral manifestations.
Zadeh et al. [36]	Healthy individuals (*n* = 21)	M1	250	10/100/1000	10	120 s	Ispta = 0.5 W/cm^2^; Isppa = 5 W/cm^2^ (Numerical simulation of BabelBrain software)	0.98 MI: 0.44–1.19	Offline: The MEP amplitude was decreased at PRF of 10/100 Hz, with effects persisting for at least 30 min and more than 60 min, respectively, while no significant change was observed at 1000 Hz. The latency remained unaltered.
Fomenko et al. [66]	Healthy individuals (*n* = 16)	M1	500	1000	30	0.1–0.5 s	Ispta = 0.69 W/cm^2^; Isppa = 2.32 W/cm^2^ (Obtained through measurements in degassed water experiments with transcranial attenuation correction)	0.1–0.3 MI: 0.19	Online: Reduces cortical excitability, enhances GABAergic inhibition, and improves reaction time.
Zhang et al. [59]	Healthy individuals (*n* = 10)	M1	500	50	2	500 ms	Ispta = 3.078–12.312 mW/cm^2^ Isppa = 0.6156–2.4624 W/cm^2^ (Measurements conducted in an ex vivo skull model)	NR MI: NR	Offline: The amplitude of MEP was significantly decreased. SICI and LICI were potentiated, while ICF was significantly attenuated. Additionally, the proportion of excitatory neurometabolites (Glx) was downregulated.
Ziebell et al. [67]	Healthy individuals (*n* = 152)	rPFC	500	40	0.5	120 s	Ispta = 199 mW/cm^2^; Isppa = 40 W/cm^2^ (In degassed water)	1.09 MI:1.86	Offline: Decrease frontal midline theta power, enhance approach-related behavior, and improve emotional state.
Badran et al. [37]	Healthy individuals (*n* = 19)	Right anterior thalamus	650	10	5	30 s	Ispta = 995 mW/cm^2^; Isppa = NR (Intracranial intensity estimated based on the FDA ultrasound attenuation model)	0.72 MI: NR	Offline: An analgesic effect is induced following the cessation of stimulation.
Cain et al. [68]	Healthy individuals (*n* = 16)	L-GP	650	10/100	5	30 s	Ispta = 720 mW/cm^2^; Isppa = 14.4 W/cm^2^ (In degassed water and numerical simulation of human CT data using k-Wave software)	1.0558 MI: NR	Online: It resulted in a significant reduction in BOLD signals within the target region, its adjacent thalamic areas, and the widespread cortical networks extensively involved in functional connectivity. Offline: It results in a significant reduction in relative perfusion levels across the entire brain.
Lee et al. [69]	DRE (*n* = 6)	SOZ	NR	100	30	600 s	Ispta ≤ 2.8 W/cm^2^; Isppa = NR (Estimation of skull attenuation using CT Images)	NR MI: 0.75	Online: Altered the SEEG power; Offline: Seizure frequency was reduced in 2 cases.

## Data Availability

No new data were created or analyzed in this study. Data sharing is not applicable to this article.

## Abbreviations

The following abbreviations are used in this manuscript:

*f*_0_	Frequency
PRF	Pulse Repetition Frequency
DC	Duty Cycle
SD	Sonication Duration
Ispta	Space-Peak-Time-Average Intensity
Isppa	Space-Peak-Pulse-Average Intensity
PD	Pulse Duration
NR	No Report
S1	Primary Somatosensory Cortex
LCN	Lateral Cerebellar Nucleus
MEP	Motor Evoked Potential
M1	Primary Motor Cortex
AD	Alzheimer’s Disease
c-Fos	Cellular Fos Protein
GAD-65	Glutamic Acid Decarboxylase, 65 kDa Isoform
BOLD	Blood Oxygenation Level-Dependent
SEP	Somatosensory Evoked Potential
V1	Primary Visual Cortex
VEP	Visual Evoked Potential
SICI	Short-Interval Intracortical Inhibition
LICI	Long-Interval Intracortical Inhibition
Glx	Glutamate/Glutamine
hMT+	Human Middle Temporal Area
ERP	Event-Related Potential
rIFG	Right Inferior Frontal Gyrus
VPL	Ventral Posterolateral Nucleus of Thalamus
ICF	Intracortical Facilitation
rPFC	Right Prefrontal Cortex
L-GP	Left Globus Pallidus
DRE	Patients with drug-resistant epilepsy
SOZ	Seizure onset zone
SEEG	stereo-electroencephalography

## Appendix A. Literature Search Methodology

To comprehensively delineate the associations between the core parameters of transcranial focused ultrasound (tFUS) and its neuromodulatory effects, this review conducted a systematic and extensive literature search across multiple electronic databases, including PubMed, Web of Science, IEEE Xplore, and Scopus, to capture pivotal studies in the field. To ensure inclusion of the most recent scientific advancements, an additional search was performed on the preprint platform bioRxiv. The search strategy integrated a combinatorial approach, combining controlled vocabulary (subject headings) with relevant free-text terms to enhance retrieval accuracy. Key search terms such as “transcranial focused ultrasound,” “tFUS,” “ultrasound neuromodulation,” and “ultrasound parameter” were strategically combined using Boolean operators (AND, OR) to maximize both recall and precision. The search spanned from the inception of each database up to 2025, ensuring comprehensive coverage of foundational and cutting-edge research.

For literature screening, predefined and rigorous inclusion and exclusion criteria were applied. Inclusion criteria required that studies: (1) investigate the neuromodulatory effects of tFUS on the central nervous system; (2) address underlying mechanisms, therapeutic efficacy, safety profiles, or optimization of technical parameters; and (3) encompass diverse study designs, ranging from animal models to human clinical trials. Exclusion criteria eliminated: (1) studies focusing exclusively on non-neuromodulatory applications of transcranial ultrasound, such as blood–brain barrier disruption or tumor ablation; and (2) publications unrelated to the scope of tFUS and neural modulation.

The application of this structured methodology enabled the selection of high-quality, relevant literature, thereby establishing a robust and credible evidentiary basis for the arguments advanced in this review.

## Appendix B. Focused Transcranial Ultrasound Stimulation Probes for Neural Modulation

The realization of transcranial focused ultrasound stimulation (tFUS) technology critically depends on its core component—the ultrasound transducer, also referred to as the transducer array—whose design directly governs the safe and effective delivery of ultrasound energy to the targeted brain region. As the technology has advanced, tFUS systems have transitioned from early single-element transducers to sophisticated multi-element phased arrays, leading to a growing prominence in the diversity of key design parameters. These parameters primarily include the number of array elements, center frequency, geometric configuration, driving electronics, and beamforming strategies [7,98].

1. The number of array elements

The number of array elements is a critical parameter that defines the classification and performance capabilities of ultrasound transducers. Early studies predominantly employed single-element concave transducers, in which the focal point was fixed by physical curvature. While these devices lack electronic steerability, they offer advantages in structural simplicity and cost-effectiveness. In contrast, modern systems increasingly rely on multi-element phased arrays, with element counts ranging from several dozen to over one thousand [99,100,101]. Such scalability in array size enables progressive improvements in beamforming precision and functional versatility:

① A 256-element configuration has been extensively adopted in landmark preclinical and clinical investigations, demonstrating an optimal trade-off between focusing accuracy and system complexity [102];

② A 512-element array provides enhanced beam steering resolution and a broader steering range, making it particularly suitable for targeting deep or peripheral brain regions with high spatial fidelity [103];

③ High-density arrays comprising 1024 or more elements represent the state of the art in transducer technology, enabling the generation of sophisticated acoustic field patterns—such as multi-focus fields—and effective correction of skull-induced wavefront distortions. These capabilities substantially improve both the spatial precision and safety profile of transcranial ultrasound neuromodulation [104].

2. Center Frequency

The choice of center frequency involves a trade-off between penetration ability (which favors lower frequencies) and spatial resolution (which benefits from higher frequencies).

① Low frequency band (0.25–0.5 MHz): A frequency around 0.25 MHz offers excellent skull penetration with relatively low attenuation and thermal effects [105]. However, it produces a larger focal spot and lower resolution, making it suitable for deep brain stimulation where precise targeting is not critical [106].

② Common frequency band (0.5–1.0 MHz): Most tFUS studies currently focus on this range (e.g., 0.5 MHz or 0.65 MHz), which balances adequate penetration depth with acceptable spatial resolution. This band has become the primary focus for clinical translation [107,108].

③ High frequency band (>1.0 MHz): Frequencies such as 1.5 MHz enable higher resolution and a smaller focal point; however, they are limited by strong attenuation and heat accumulation in the skull, thus mainly applied in areas with thinner skull bones or in animal models like mice [109].

3. Geometric Shapes

The geometric configuration of transducers plays a critical role in determining the spatial distribution and controllability of their acoustic fields.

① Spherical concave designs are widely adopted in single-element and early phased array transducers, where the radius of curvature (e.g., 64 mm or 90 mm) defines the location of the natural focal point, enabling electronic focusing to further refine the focal position [110,111];

② Planar arrays lack an inherent focal point and rely entirely on electronic beamforming to achieve focusing. While they offer advantages in terms of simpler fabrication and ease of integration into wearable platforms such as helmets, their performance deteriorates significantly when steering the beam to large angles off-axis [112];

③ Hemispherical or conformal configurations arrange the transducer elements on a curved surface that matches the contour of the head, thereby substantially extending the accessible range of acoustic steering. This design enables near-complete coverage of the brain and is considered a pivotal advancement toward achieving comprehensive, non-invasive brain-targeted stimulation [113].

4. Driving Electronics System

The driving electronics system functions as the control center of the phased array, responsible for supplying each array element with independent and precise excitation signals. A typical system consists of the following components:

① A main controller (e.g., an FPGA), which executes beamforming algorithms and generates high-precision timing control signals.

② Independent high-voltage amplifiers and switching circuits for each channel, capable of delivering electrical pulses ranging from tens to hundreds of volts to drive the piezoelectric transducers [114].

③ Advanced systems often incorporate real-time monitoring and feedback mechanisms. For example, by monitoring electrical impedance at the transducer end, channel failures or coupling issues can be detected, thereby ensuring operational safety and reliability [115].

5. Beamforming Technology

Beamforming is the core technique that enables phased arrays to achieve electronic focusing and dynamic beam steering. It synthesizes a desired acoustic field distribution by precisely controlling the time delays (i.e., phase shifts) and amplitude weights of the signals emitted from individual array elements.

① Model-based beamforming is a widely adopted approach. It involves acquiring individual CT or MRI head scans and combining them with acoustic simulations—such as the angular spectrum method or finite-element analysis—to calculate the optimal delay sequences required for ultrasound to traverse the specific cranial structures. This compensates for skull-induced sound-speed variations and wavefront distortions [116,117], thereby significantly improving focusing accuracy.

② Waveguide-based beamforming represents an emerging alternative. This method employs implantable or wearable acoustic reference beacons (e.g., micro-sensors) to directly measure the transmission function from each transducer element to the target point, from which the optimal driving parameters are inversely derived [118]. By circumventing the modeling process and its associated assumptions, this approach reduces model-related inaccuracies and offers greater potential for theoretical precision [119].

## Appendix C. The Link Between Brain Rhythms and Underlying Neuronal Excitation-Inhibition

**Rhythms**	**Frequency Band (Hz)**	**The Significance of EEG**	**The Potential Connection with Neural Modulation (Excitation/Inhibition) (Based on MEP Evidence, All Studies Were Conducted on Healthy Individuals)**
δ	0.5–4 [120]	The hallmark of deep non-rapid eye movement sleep (slow-wave sleep) [120]. These delta oscillations are critically involved in synaptic homeostasis regulation and support system-level memory consolidation processes [121].	During deep sleep characterized by prominent delta wave activity, MEP amplitude is markedly reduced or often fails to be elicited, indicating a profound inhibitory state of the cerebral cortex and corticospinal pathways [122].
θ	4–8 [120]	The midline prefrontal theta oscillation is strongly associated with cognitive control, conflict monitoring, as well as the encoding and maintenance processes of working memory [123,124]. Such oscillatory activity exhibits enhancement during tasks demanding high cognitive effort and sustained attention.	During inhibitory control tasks, prefrontal theta oscillatory activity is significantly enhanced; specific phases of the theta rhythm in the sensorimotor cortex may correspond to time windows of reduced neural excitability, indicating its critical role in top-down inhibitory regulation [123,124]. Furthermore, the modulatory effect of theta phase on TMS-induced neural plasticity further substantiates the functional relevance of theta oscillations in phase-dependent excitability regulation [125].
α	8–13 [120]	It is most prominent in the occipital lobe under conditions of wakefulness, relaxation, and closed eyes, and is often recognized as the “cortical idling” or “inhibitory control” rhythm [120].	The alpha rhythm subserves functional cortical inhibition, with elevated power correlating with the gating of irrelevant sensory inputs [126,127]. Within the sensorimotor cortex, the power of the α(μ) rhythm exhibits a negative correlation with MEP amplitude [128], while its phase can transiently modulate cortical excitability [129].
β	13–30 [120]	It is associated with motor preparation, execution, maintenance, and cognitive control, and is recognized as a “status quo maintenance” signal [130,131].	Elevated β power (e.g., during resting state or post-motor “β rebound”) is accompanied by reduced MEP amplitude [132]. Its oscillatory phase can also modulate excitability [129], suggesting that enhanced β activity may inhibit the initiation of novel movements and stabilize the existing state.
γ	30–45 [120]	It has been associated with high-order processes including feature binding, selective attention, conscious perception, and memory retrieval [133,134].	The augmentation of γ activity (e.g., during the motor preparation period) is accompanied by an increase in MEP amplitude [135], indicating that it serves as a physiological marker for the enhanced excitability and information integration of local cortical networks.
